# *Sclerocarya birrea caffra* nut meal as a substitute to soyabean meal: Effects on growth performance, feed intake and utilisation and viscera macromorphometry of blue-helmeted guinea fowl

**DOI:** 10.1016/j.vas.2022.100254

**Published:** 2022-06-14

**Authors:** Thandanani Z. Nkwanyana, Busisani Lembede, Eliton Chivandi

**Affiliations:** School of Physiology, Faculty of Health Sciences, University of the Witwatersrand, 7 York Road, Parktown, Johannesburg, 2193, South Africa

**Keywords:** Numida meleagris, Sclerocarya birrea, Non-conventional feed ingredients, Growth performance, Feed utilization efficiency, GF, Guinea fowl, DP, Dietary protein, SBM, Soyabean meal, MNM, Marula nut meal, CP, Crude protein, FI, Feed intake, TBM, Terminal body mass, BMG, Body mass gain, ADG, Average daily gain, FCR, Feed conversion ratio, SSA, sub - Saharan Africa, WRAF, Wits Research Animal Facility, GIT, Gastrointestinal tract

## Abstract

Imported soyabean meal (SBM) is the major dietary protein (DP) source for the sub-Saharan African poultry industry making poultry production costly. Therefore, alternative locally available DP sources are required. We evaluated the potential of locally available Marula nut meal (MNM) to substitute SBM in Guinea fowl (GF) diets by determining its effects on growth, feed intake (FI) and utilisation and viscera macromorphometry. Five grower diets wherein, on a CP basis, MNM substituted SBM at 0, 25, 50, 75 and 100% were formulated. Thirty-eight 4-week-old keets (*n* = 7 – 8), each individually housed in a cage, were randomly assigned to grower diets, and fed for 5 weeks and then transferred to corresponding finisher diets and fed for 3 weeks. Induction and weekly body mass, daily FI, and terminal body mass (TBM), body mass gain (BMG), average daily gain (ADG) and feed conversion ratio (FCR) were determined. On slaughter, viscera masses, small and large intestines lengths, tibiae and femora indices were determined. In week 2 of the grower phase GF fed diet 3 (50% MNM CP) had the highest weekly BMG and ADG (*P* < 0.05) and in week 5 GF fed diet 5 (100% MNM CP) had the highest FI (*P* < 0.05). Dietary MNM did not affect the GF's BMG, ADG, FI and FCR during weeks 1, 3 and 4 of the grower phase. In week 3 of the finisher phase GF fed diet 3 (50% MNM CP) had the highest (*P* < 0.05) FCR. Dietary MNM had no effect (*P* > 0.05) on the trial BMG, ADG and FI of the GF but GF reared on grower and finisher diets 3 (50% substitution of SBM CP) had the highest (*P* < 0.05) FCR. MNM had no effect on tibiae and femora masses, lengths, and mass: length ratios and viscera macromorphometry of the GF. We conclude that MNM can, on a CP basis, substitute SBM, in GF grower and finisher diets at 25%, 75% and 100% without compromising growth, FI and utilization and viscera of GF.

## Highlights

• Marula nut meal can be used as a replacer of soyabean meal in guinea fowl feeds.
• Use of other tree seeds as dietary protein sources in poultry feeds needs further exploitation.
• Marula nut meal does not affect the viscera macromorphometry and feed utilisation in guinea fowl.

## 1 Introduction

Broiler chicken meat is relatively more affordable when compared to red meats such as beef and lamb (Delport, Louw, Davids, Vermeulen & Meyer, 2017). Additionally when compared to red meats chicken meat has a lower fat and cholesterol content (Komprda, Zelenka, Fajmonovaa, Bakaj & P, 2003) but higher protein content (Barroeta, 2015) which makes it a more healthful product. This has spawned a huge demand for the meat. However, the production of improved broiler and pullet chicken breeds is associated with high housing, feed, and veterinary costs (Gale & Arnade, 2015; Matthews & Sumner, 2015) that hinder rural communities, where animal-derived protein malnutrition is highest, from producing them (Kolahdooz, Spearing & Sharma, 2013). There is a dire need to consider alternative poultry species that can be successfully produced by rural communities. Guinea fowls (*Numida meleagris*), whose production has increased in organic agricultural systems (Eleroğlu, et al., 2016; Abdul-Rahman & Adu, 2017), offer potential as an alternative to improved chicken breeds in rural settings. Compared to improved chicken breeds, Guinea fowl (GF) are hardier. Their meat is lean, has a high protein (25.6%) content (Musundire, Halimani & Chimonyo, 2017) making it a nutritionally healthier product when compared to broiler chicken meat (Mir, Rafiq, Kumar, Singh & Shukla, 2017) which has a higher fat content (1.2%) (Musundire et al., 2017). These attributes make GF more suited for production by resource-poor rural and or smallholder farmers (Saina, 2005).

Nutritionally balanced diets are a necessity in ensuring optimal growth performance, feed economy, bird health and product (eggs and meat) quality. Plant and animal-derived protein sources are used as DP in the formulation of poultry diets (Denton, Coon, Pettigrew & Parsons, 2005). The dietary protein component in poultry diets, chiefly soyabean meal (SBM) is the most expensive dietary ingredients which significantly contributes to the high cost of poultry feed (Beski, Swick & Iji, 2015). In South Africa soyabean production is not enough to produce the required amounts of SBM by the South African poultry industry (Dlamini, Tshabalala & Mutengwa, 2016) due to competition for SBM between human and the poultry industry's needs. To mitigate the high cost imposed on poultry production due to dependency on imported SBM, there is a dire need to search and develop alternative DP sources that can be used to support GF production by rural households. Such alternative DP sources will help attenuate the shortage of animal-derived protein in rural communities. Tree seeds from indigenous fruit bearing trees in sub-Saharan Africa (SSA) can be exploited as DP sources in poultry feeds. Marula (*Sclerocarya birrea caffra*), an indigenous fruit bearing tree, whose seed has a high content of ascorbic acid (2960 mg/100 g) (Nitcheu Ngemakwe, Remize, Thaoge & Sivakumar, 2017; Ojewole, Mawoza, Chiwororo & Owira, 2010), calcium (51 mg/100 g) and phosphorus (19 mg/100 g)( (Abdulla, Loh, Akit, Sazili & Foo, 2016; Stadlmayr, Charrondière, Eisenwagen, Jamnadass & Kehlenbeck, 2013) all key nutrients in poultry nutrition, is widely distributed throughout SSA (Mokgolodi, You-fang, Setshogo, Chao & Yu-jun, 2011). While the fruit pulp of *Sclerocarya birrea caffra* contains 84% carbohydrate (Wairagu, Kiptoo & G, 2013) the kernels of Nigerian *S. birrea caffra* provenance contain 36.7% crude protein (Mariod & Abdelwahab, 2012) and full fat South African *S. birrea caffra* kernels contain 28% crude protein (Wynberg et al., 2012). The mechanically defatted *S. birrea caffra* kernel meal contains 33–39% crude protein (Malebana, Nkosi, Erlwanger & Chivandi, 2018). These favourable nutritional attributes make *S. birrea caffra* kernel meal a potential DP and energy source in poultry feeds. Previous studies have shown that Marula nut meal (MNM) has been successfully utilised as a DP source in sheep (Habibu et al., 2016), cattle (Mdziniso, Dlamini, Khumalo & Mupangwa, 2016), broiler chicken feeds (Mdzinisoet al., 2016) and Japanese quail (Mazizi, Moyo, Erlwanger & Chivandi, 2019). Despite the reported successful use of the MNM as a DP source in the feed different livestock and poultry species, its potential has not been evaluated in Guinea fowl. The gastrointestinal tract (GIT) microbiota composition differs between and amongst bird species and it (GIT microbiota composition) impacts nutrient digestion and absorption in birds. We therefore evaluated the effects of a graded substitution of SBM with MNM as a DP source in Guinea fowl grower and finisher diets on GF growth performance, feed intake (FI) and utilisation and viscera macro-morphometry.

## 2 Materials and methods

### 2.1 Study site and animal ethical clearance

The study was conducted at the Wits Research Animal Facility (WRAF) after obtaining ethical clearance from the University of the Witwatersrand Animal Research Ethics Committee, South Africa (Ethics clearance number: 2018/07/31/B). Assays on tissue samples collected from the GF at study termination were done in the Wits School of Physiology laboratories.

### 2.2 Marula nut meal processing and other feed ingredients

Mechanically defatted MNM was procured from Mhlala Development Centre, a medium scale Marula oil extraction company in Bushbuckridge, Limpopo, South Africa. Due to high residual oil content of the mechanically defatted MNM, the meal was further defatted by solvent extraction with hexane. In summary, each 40 kg batch of the mechanically defatted MNM in a cotton bag was steeped, for 48 h, in 400 litres of 99.9% hexane in an 800-litre stainless steel tank equipped with a drainage tap (Trade All Africa Engineering & Industrial Pty Ltd, South Africa) immediately followed by draining the oil-laden hexane through opening a tap at the bottom side of the tank. The hexane extracted MNM was then spread onto clean plastic sheets to dry for 24 h under room temperature. Following drying, the hexane extracted MNM was packaged into jute bags and stored at room temperature till diet formulation in combination with other feed ingredients. Yellow maize, wheat bran and SBM were purchased from Obaro (Pty) Ltd, Pretoria, South Africa, and canola oil and iodated salt were purchased from Makro (Pty) Ltd, Johannesburg, South Africa. The vitamin-mineral premix was sourced from Trouw Nutrition, Edenvale, South Africa.

### 2.3 Housing and management

Thirty-eight-day-old GF (*Numida meleagris*) keets were sourced from Dominion Outfitters, Durban, KwaZulu-Natal, South Africa. On arrival at the WRAF, the day-old keets were treated with 1 ml/L Enrovet oral solution (Kyron Laboratories Pvt Ltd, Johannesburg, South Africa) in drinking water for 3 days grown to 4 weeks of age on a commercial broiler starter diet before commencement of the dietary interventions. During this pre-trial period, the keets were housed in a pen under a deep litter system where clean wood shavings were used for bedding which (bedding) was changed once weekly. During the 4-week pre-trial preparatory period, a 12-hour light regimen was followed with lights on at 06h00 and lights off at 18h00. Infrared lamps were used to provide supplementary heat and room temperature was kept at 24 °C. The keets had *ad libitum* access to a commercial broiler chicken starter feed throughout the pre-trial period. At 4-week-old, at the commencement of the feeding trial, the 38 GF keets were moved from the group pen housing to individual housing of each keet in a cage (0.60 m length x 0.60 m width x 0.80 m height) equipped with a feeding and watering trough. The cage design and placement allowed visual, smell and sound visual contact to mitigate stress. Each bird had *ad libitum* access to its respective dietary treatment and clean drinking water. The temperature of the room where the keets were individually housed in cages was maintained at 24 °C as recommended by Mushtaq et al. (2013) and a 12-hours light cycle (with lights on from 06h00 to 18h00) was practiced throughout the feeding trial.

### 2.4 Diets and formulation

The grower and finisher diets were formulated such that MNM substituted SBM on a crude protein (CP) basis at 0, 25, 50, 75 and 100% for diet 1 through to 5, respectively. These diets were formulated to meet the nutritional requirements of GF at the grower and finisher growth phases recommended by Ensminger, Oldfield and Heinemann (1990). The ingredient and chemical nutrient composition of the grower and finisher diets are shown in Tables 1 and 2.

### 2.5 Experimental design

Thirty-eight 28-day old unsexed (*n* = 7 – 8) GF keets following a 2-day habituation to individual cage housing were, in a completely randomized design, allocated to the grower diets in which MNM replaced SBM's CP contribution to the diets on a graded levels at 0%, 25%, 50%, 75% and 100%, for diets 1 through to 5, respectively. The GF keets were reared on the grower diets for 5 weeks and then transferred onto corresponding finisher diets in which the MNM replace the SBM's CP contribution to the finisher diets also at 0%, 25%, 50%, 75% and 100%, for finisher diets 1 through to 5, respectively. The GF were fed respective finisher diets for 3 weeks.

### 2.6 Measurements and computations

Induction and weekly body masses were measured using an electronic balance (Snowrex EQ-1200, Snowrex International Company, Taipei, Taiwan). Daily feed intake (FI) was determined. Body mass gain (BMG) and average daily gain (ADG) were computed from the induction and weekly body mass data and feed conversion ratio (FCR) was computed from the body mass gain and feed intake data.

### 2.7 Terminal procedures, sample collection and measurements

At the end of the finisher phase of the feeding trial, the GF were subjected to a 4-hour fast but with access to clean drinking water and then their terminal body mass (TBM) measured immediately thereafter. Following measurement of the TBM, each GF was humanely killed by exsanguination using a guillotine. After feather plucking, a midline incision was made on each carcass and gastrointestinal tract [GIT (proventriculus, ventriculus, small and large intestines and caeca)] and GIT accessory (liver and pancreas) organs were dissected out and the mass and length (for the small and large intestines) determined using and electronic balance and a ruler attached on the dissection board, respectively. The left femur and tibia from each carcass were excised and soft tissues removed. The bones were dried in an oven (Salvis ®, Salvis Lab, Switzerland) at 50 °C for 5 days to constant weight. Thereafter, the dried bones were weighed on a digital scale and the length of each bone measured using an electronic digital Vernier calliper (SDP-S-ETP-1001, Major Tech,

Johannesburg, South Africa). Tibia length was measured from the proximal end to the distal end and the width at the medial diaphysis.

### 2.8 Statistical analysis

All parametric data are expressed as mean ± SD. The data were analysed using GraphPad Prism 5 software (Graph-Pad Software Inc., San Diego, CA, USA). The weekly body masses and feed intake of birds within groups were analysed using repeated measures ANOVA. A one-way ANOVA was used to analyse all other multiple group parametric data. Tukey's *post-hoc* test was used to compare means. Statistical significance was set at *P* < 0.05.

### 2.9 Results

No mortality was recorded during the course of the feeding trial. Table A.3 shows the effect dietary MNM on the growth performance, feed intake, feed utilisation efficiency of the GF and Table A.4 shows the effect of dietary MNM on GIT and GIT accessory organs macromorphometry. The induction body mass of the GF was similar across treatment groups and the TBM of the GF was similar across dietary treatments (*P* > 0.05), but the GF grew significantly (*P* < 0.05) from the induction to the termination of the feeding trial. In week 2 of the grower phase GF feed diet 3 containing 50% MNM CP had the highest (*P* < 0.05) weekly BMG and ADG and in week 5 GF fed diet 2 and 3 containing 25% and 50% MNM CP, respectively, had the highest (*P* < 0.05) FI. Dietary MNM did not affect the GF's BMG, ADG, FI and FCR during weeks 1, 3 and 4 of the grower phases. In week 3 of the finisher phase GF fed diet 3 containing 50% MNM CP had the highest (*P* < 0.05) FCR. Dietary MNM had no effect (*P* > 0.05) on the trial (grower and finisher phases) BMG, ADG and FI of the GF but GF reared on grower and finisher diets 3 (50% substitution of SBM CP) had the highest (*P* < 0.05) FCR.

Dietary MNM had no effect (*P* > 0.05) on the masses of proventricular, ventriculi, small and large intestines, liver, and pancreas as well as the lengths of the small and large intestines of the GF. The effect of dietary MNM on the femora and tibia indices of the GF are presented in Table A.5. Dietary MNM did not affect (*P* > 0.05) femora and tibia mass, length, and mass: length ratio of the GF birds.

## 3 Discussion

Growth performance and feed utilisation efficiency are critical determinants of profitability of any poultry production enterprise (Carvalho, Zilli, Mendes, Morello & Bonamigo, 2015). An enhanced growth performance allows for the untying of space, making it possible to have many batches of the birds being grown to slaughter mass. High feed utilisation efficiencies typified by the attainment of the expected growth in body mass terms for less feed consumed (Burlingame, 2004) help reduce the feed cost contribution to total production costs thus increase profitability. Our findings show that in week 2 of the grower growth phase, GF fed diet 3 (50% MNM CP replacement of SBM's CP contribution) had higher weekly BMG and ADG compared to counterparts fed other diets and in week 5 of the grower phase GF fed diets 2 (25% MNM CP replacement of SBM's CP contribution) and 3 (50% MNM CP replacement of SBM's CP contribution), respectively had higher FI compared to the rest but with no differences in the GF's FCR across dietary treatments. While we report no differences in weekly BMG, ADG and FI in the finisher growth phase, in week 3 of this growth phase the FCR of the GF fed diet 3 was higher compared to that of the rest. Additionally, and of note is that the GF's trial BMG, ADG total FI were similar across dietary treatments but with the trial FCR for GF fed diet 3 being the highest. Our findings show that in the second week of the grower growth phase GF fed diet 3 (50% MNM CP replacement of SBM's CP contribution) lagged in growth performance compared to counterparts fed diets 1, 2, 4 and 5 demonstrated by their lower weekly BMG but with a higher FCR. However, the similarity in the BMG, ADG in weeks 4 and 5 of this growth (grower) phase indicate compensatory growth. Of interest is the higher FI in week 5 of the grower growth phase by GF feed diets 2 and 3 (25% and 50% dietary substitution of SBM with MNM on a CP basis, respectively) but which (higher FI) is not translated to higher BMG and ADG demonstrating inefficient feed utilisation efficiency by the GF during the fifth week of the grower phase. Similarities in the trial (combined grower and finisher phase) BMG, ADG, FI and FCR of GF reared on diets 1,2, 4 and 5 demonstrate that MNM can be used to replace 25%, 75% and 100%, respectively of SBM's CP contribution to the diet without compromising growth performance (as measured body mass-based indices), FI and utilisation efficiency. It can be inferred from our findings that substitution of SBM's dietary CP contribution at 50% results in poor feed utilisation efficiency as demonstrated by the high trial FCR of GF feed diet 3. We acknowledge that it is challenging to give a solid reason for this observed poor feed utilisation when replacing 50% of SBM's contribution with MNM but it can be speculated that the poor feed utilisation efficiency might have resulted from negative associative effects from feed ingredients at this level of substitution.

Though body mass-based indices of growth performance are still of value, it must be noted that these indices are affected by gut fill (Steyn, Casey & Jansen van Rensburg, 2012) and hydration status (Popkin & Rosenberg, 2011) of the birds, they are thus not accurate measures of growth performance. The mass, length, and bone mass to length ratio of tibiae and femora are a better proxy for the evaluation of growth performance since growth of these antigravity bones respond to growth hormone in a dose-dependant manner (Melin, Bergmann & Russell, 2005). Use of indices derived from these anti-gravity (long) bones give a more accurate evaluation of dietary interventions on growth performance (Lourenço, Villamor, Augusto & Cardoso, 2012). Findings from our study show similarities in femora and tibiae masses, lengths, and mass: length ratios of the GF across dietary treatments suggesting that MNM can substitute SBM as a dietary protein source in grower and finisher GF diets without compromising growth performance of GF

Feed ingredients and diet composition are fundamental factors that affect growth (de Quelen, Brossard, Wilfart, Dourmad & Garcia-Launay, 2021), development and function of the gastrointestinal tract (Celi et al., 2017). de Vries (2015) reports that moderate amounts of dietary fibre in poultry feeds to be beneficial for GIT development, health and function translating to enhanced nutrient digestion and absorption and increased growth performance. Additionally and interestingly, the lipid profile of lipid-rich broiler chicken diets, has been shown to mirror the adipose tissue of the chicken (Wang, Kim, Cline & Gilbert, 2017; Zollitsch, Knaus, Aichinger & Lettner, 1997) which (adipose tissue) accretion can impact body mass. *Moringa oleifera* leaf meal (Alagbe, 2019) and Baobab seed meal contain coarse fibre (Svihus, 2014): use of these meals as dietary components in poultry diets positively impacts poultry performance by improving digestion and nutrient absorption (Alagbe, 2017; Gunya, 2016; Nkukwana et al., 2014). Our findings show that dietary MNM had no effect on the GIT (proventriculus, ventriculus, small and large intestines, caeca) and GIT accessory organ (liver and pancreas) masses as well as the lengths of the small and large intestines of the GF suggesting that the MNM neither compromised nor improved GIT accessory organs growth, development, and digestive function. These findings are consistent to Mazizi et al. (2019), who did not find significant differences on the viscera of Japanese quail fed graded MNM. However, they are contrary to (Mazizi et al., 2019) who also reported caecum (relative to body mass%) from quail fed diet 4 (75% MNM CP replacement of SBM's CP contribution) to be lighter than that of quail fed diets 3. Similarities in the terminal body masses of GF across dietary treatments and similarities in trial BMG, ADG and FI and FCR of GF reared on grower and finisher diets 1, 2, 4 and 5, give credence to our assertion that dietary MNM did not compromise the digestive and absorptive function of the gastrointestinal tracts of the GF.

## 4 Conclusion

Based on our findings, we conclude that MNM can be used to substitute of SBM's CP contribution to GF grower and finisher diets at 25%, 75% and 100% without compromising the growth performance. While the MNM effectively replaced SBM in GF diets, future studies should perform a cost benefit analysis to determine the economic benefit of using MNM in place of SBM. Moreover, caution needs to be taken at 50% MNM inclusion, as it evidently reduced feed intake of the GF.

## Declaration of Competing Interests

Thandanani Zola Nkwanyana reports financial support was provided by National Research Fund.

## Figures and Tables

**Table:** 

			**Grower**		
**Ingredients (g/kg)**	**Diet 1**	**Diet 2**	**Diet 3**	**Diet 4**	**Diet 5**
Soyabean meal (45%)	369.34	280.46	187.69	94.37	0.00
Yellow maize meal	491.85	562.57	518.42	572.57	623.01
Marula nut meal	0.00	65.57	131.60	198.54	272.52
Wheat bran	91.08	46.11	120.35	93.10	62.30
Soyabean oil	11.84	7.01	2.59	0.00	0.00
Limestone	20.95	21.21	21.29	21.41	22.04
*DL*-Methionine, 99%	2.19	1.84	1.30	0.93	0.48
*L*-Lysine HCL 98.5%	0.00	2.31	3.80	6.05	8.15
Di-calcium phosphate	3.64	3.69	3.70	3.72	1.92
Salt	4.55	4.61	4.63	4.66	4.79
Vitamin/mineral premix*	4.55	4.61	4.63	4.66	4.79
**Total**	**1000**	**1000**	**1000**	**1000**	**1000**
***Chemical composition (%)***					
Dry matter	90.00	89.15	88.88	88.64	86.37
Crude protein	21.94	21.93	22.80	22.95	22.85
Ether extract	3.67	3.63	3.63	3.84	4.21
Calcium	1.03	1.00	0.98	0.95	0.88
Phosphorus	0.41	0.46	0.53	0.58	0.59
Gross energy	16.89	16.61	16.51	16.40	16.01

**Table:** 

			**Finisher**		
**Ingredients (g/kg)**	**Diet 1**	**Diet 2**	**Diet 3**	**Diet 4**	**Diet 5**
Soyabean meal (45%)	235.32	176.98	117.70	58.66	0.00
Yellow maize meal	583.58	599.38	616.73	633.57	652.18
Marula nut meal	0.00	41.37	82.54	123.42	163.34
Wheat bran	141.19	141.59	141.23	140.79	139.75
Soyabean oil	0.00	0.00	0.00	0.00	0.00
Limestone	21.65	23.13	23.54	24.40	24.69
DL-Methionine, 99%	3.20	2.93	2.73	2.44	2.24
L-Lysine HCL 98.5%	1.41	2.83	3.77	4.97	6.15
Di-calcium phosphate	5.65	3.78	3.77	3.75	3.73
Salt	3.29	3.30	3.30	3.29	3.26
Vitamin/mineral premix*	4.71	4.72	4.71	4.69	4.66
**Total**	**1000**	**1000**	**1000**	**1000**	**1000**
***Chemical composition (%)***					
Dry matter	87.27	87.17	87.54	87.96	88.77
Crude protein	16.97	17.19	17.41	17.64	17.92
Ether extract	2.74	3.02	3.31	3.60	3.91
Calcium	1.02	1.00	1.00	1.02	1.02
Phosphorus	0.41	0.41	0.44	0.48	0.51
Gross energy	15.72	15.71	15.76	15.82	15.96

**Table:** 

			**Dietary treatments**				
**Parameter**	**Week**	**Diet 1**	**Diet 2**	**Diet 3**	**Diet 4**	**Diet 5**	**Significance**
Induction body mass (g)		241 ± 50.5^a^	288 ± 62.9 ^a^	291 ± 68.1 ^a^	212 ± 95.6 ^a^	241 ± 95.9 ^a^	ns
Terminal body mass (g)		869 ± 88.1^a^	954 ± 115^a^	968 ± 233^a^	771 ± 222^a^	809 ± 185^a^	ns
**Grower phase**							
BMG (g)	1 2 3 4 5	35.0 ± 11.4^a^ 80.4 ± 19.4^a^ 58.9 ± 26.4 ^a^ 119 ± 26.8 ^a^ 77.0 ± 25.5 ^a^	52.9 ± 13.8 ^a^ 74.4 ± 27.4^a^ 89.0 ± 49.4 ^a^ 87.0 ± 30.6 ^a^ 104 ± 52.4 ^a^	50.0 ± 15.7 ^a^ 107 ± 38.7^b^ 93.8 ± 39.4 ^a^ 83.5 ± 40.5 ^a^ 97.6 ± 36.4 ^a^	29.1 ± 15.3^a^ 63.3 ± 20.9^a^ 65.1 ± 33.8 ^a^ 96.0 ± 37.8 ^a^ 78.4 ± 45.3 ^a^	47.8 ± 23.3^a^ 67.3 ± 31.1^a^ 43.4 ± 29.6 ^a^ 127 ± 62.5 ^a^ 90.8 ± 26.2 ^a^	ns * ns ns ns
							
ADG (g/d)	1 2 3 4 5	5.00 ± 1.63^a^ 11.5 ± 2.77^a^ 8.41 ± 3.77^a^ 16.9 ± 3.82^a^ 11.0 ± 3.624^a^	7.55 ± 1.97^a^ 10.6 ± 3.91^a^ 12.7 ± 7.05^a^ 12.4 ± 4.37^a^ 14.8 ± 7.48^a^	7.14 ± 2.25^a^ 15.2 ± 3.91^b^ 13.4 ± 5.63 ^a^ 11.9 ± 5.78^a^ 13.9 ± 5.20^a^	4.16 ± 2.16^a^ 9.04 ± 2.99^a^ 9.31 ± 4.82^a^ 13.7 ± 5.41^a^ 11.2 ± 6.47^a^	6.82 ± 3.33^a^ 9.61 ± 4.44^a^ 6.95 ± 3.67^a^ 18.0 ± 8.63^a^ 13.0 ± 3.74^a^	ns * ns ns ns
							
FI (g)	1 2 3 4 5	192 ± 23.2^a^ 145 ± 21.1^a^ 181 ± 27.7^a^ 200 ± 25.2^a^ 202 ± 17.7^a^	215 ± 39.5^a^ 172 ± 25.4^a^ 190 ± 29.7^a^ 219 ± 28.2^a^ 253 ± 46.4^b^	212 ± 41.8^a^ 178 ± 28.1^a^ 204 ± 39.7^a^ 224 ± 43.2^a^ 265 ± 52.5^b^	181 ± 62.6^a^ 133 ± 42.4^a^ 164 ± 40.5^a^ 178 ± 50.0^a^ 182 ± 54.1^a^	203 ± 39.2^a^ 142 ± 27.4^a^ 174 ± 34.3^a^ 206 ± 34.7^a^ 205 ± 43.9^a^	ns ns ns ns **
							
FCR	1 2 3 4 5	6.15 ± 2.58^a^ 1.89 ± 0.47^a^ 3.52 ± 1.31^a^ 1.77 ± 0.46^a^ 2.49 ± 1.03^a^	4.22 ± 0.84^a^ 2.82 ± 1.83^a^ 2.44 ± 0.67^a^ 3.18 ± 2.38^a^ 2.60 ± 0.91^a^	4.63 ± 1.87^a^ 1.92 ± 0.85^a^ 3.03 ± 2.57^a^ 7.35 ± 14.0^a^ 2.99 ± 0.935^a^	7.72 ± 4.15^a^ 2.31 ± 1.08^a^ 3.25 ± 2.39^a^ 2.28 ± 1.59^a^ 3.05 ± 2.90^a^	5.64 ± 3.95^a^ 2.53 ± 1.14^a^ 7.44 ± 7.26^a^ 1.98 ± 0.95^a^ 2.41 ± 0.74^a^	ns ns ns ns ns
**Finisher phase**							
BMG (g)	6 7 8	70.7 ± 26.7^a^ 73.7 ± 37.2^a^ 68.9 ± 35.2^a^	74.5 ± 45.5^a^ 71.5 ± 28.3^a^ 99.2 ± 65.4^a^	103 ± 34.3^a^ 62.9 ± 34.0^a^ 45.6 ± 42.0^a^	63.9 ± 34.9^a^ 80.0 ± 49.4^a^ 78.5 ± 54.6^a^	77.9 ± 21.8^a^ 54.6 ± 40.4^a^ 77.6 ± 64.4^a^	ns ns ns
ADG (g/d)	6 7 8	10.1 ± 3.82^a^ 10.5 ± 5.32^a^ 9.08 ± 4.91^a^	10.6 ± 6.50^a^ 10.2 ± 4.04^a^ 12.3 ± 8.07^a^	14.7 ± 4.90^a^ 8.98 ± 4.85^a^ 5.47 ± 5.47^a^	9.12 ± 4.99^a^ 11.4 ± 7.05^a^ 10.8 ± 8.34^a^	11.1 ± 3.12^a^ 7.80 ± 5.77^a^ 11.0 ± 9.83^a^	ns ns ns
FI (g)	6 7 8	245 ± 20.1^a^ 266 ± 24.6^a^ 296 ± 32.8^a^	271 ± 43.7^a^ 291 ± 47.8^a^ 311 ± 38.5^a^	293 ± 51.1^a^ 320 ± 59.6^a^ 344 ± 65.2^a^	235 ± 52.3^a^ 264 ± 56.7^a^ 293 ± 62.2^a^	248 ± 49.2^a^ 278 ± 47.8^a^ 303 ± 40.7^a^	ns ns ns
FCR	6 7 8	3.86 ± 1.30^a^ 3.71 ± 1.62^a^ 6.64 ± 5.01^a^	−1.85 ± 15.2^a^ 5.52 ± 4.87^a^ 3.92 ± 1.63^a^	3.03 ± 0.69^a^ 7.12 ± 5.38^a^ 29.8 ± 32.6^b^	7.44 ± 9.98^a^ 4.92 ± 4.18^a^ 9.46 ± 13.1^a^	3.31 ± 0.86^a^ 2.71 ± 5.18^a^ 1.48 ± 5.15^a^	ns ns *
**Trial (week 1–8)**							
BMG (g)		578 ± 101^a^	639 ± 67.5^a^	635 ± 152^a^	552 ± 205^a^	585 ± 139^a^	ns
ADG (g)		10.3 ± 1.80^a^	11.4 ± 1.21^a^	11.3 ± 2.72^a^	9.85 ± 3.67^a^	10.4 ± 2.48^a^	ns
FI (g)		1726 ± 99.2^a^	1921 ± 270^a^	2039 ± 370^a^	1631 ± 388^a^	1758 ± 288^a^	ns
FCR		30.0 ± 8.34^a^	22.8 ± 17.7^a^	59.9 ± 37.2^b^	40.4 ± 24.9^a^	27.5 ± 9.87^a^	*

**Table:** 

		**Dietary treatments**					
**Parameter**	**Diet 1**	**Diet 2**	**Diet 3**	**Diet 4**	**Diet 5**	**Significance**	
**Liver (g)**	13.6 ± 2.78^a^	14.5 ± 2.98^a^	14.8 ± 2.56^a^	11.1 ± 3.56^a^	11.6 ± 3.42^a^	ns	
(% body mass)	1.57 ± 0.23^a^	1.52 ± 0.21^a^	1.60 ± 0.41^a^	1.44 ± 0.48^a^	1.51 ± 0.48^a^	ns	
**Pancreas (g)**	1.43 ± 0.53^a^	1.44 ± 0.32^a^	1.56 ± 0.17^a^	1.36 ± 0.63^a^	1.25 ± 0.46^a^	ns	
(% body mass)	0.16 ± 0.05^a^	0.15 ± 0.03^a^	0.17 ± 0.06^a^	0.17 ± 0.05^a^	0.16 ± 0.06^a^	ns	
**Proventriculus (g)**	2.29 ± 0.75^a^	2.13 ± 0.954^a^	5.44 ± 9.55^a^	2.14 ± 1.07^a^	2.19 ± 0.70^a^	ns	
(% body mass)	0.26 ± 0.07^a^	0.23 ± 0.10^a^	0.56 ± 0.92^a^	0.26 ± 0.08	0.28 ± 0.12^a^	ns	
**Ventriculus (g)**	23.7 ± 6.29^a^	26.4 ± 4.66^a^	26.8 ± 9.40^a^	20.4 ± 10.5^a^	23.0 ± 9.86^a^	ns	
(% body mass)	2.71 ± 0.52^a^	2.76 ± 0.23^a^	2.95 ± 1.33^a^	2.55 ± 1.13^a^	2.89 ± 1.24^a^	ns	
**Small intestines (g)**	12.1 ± 1.95^a^	14.6 ± 2.91^a^	14.6 ± 3.49^a^	11.9 ± 3.08^a^	13.9 ± 3.10^a^	ns	
(% body mass)	1.39 ± 0.13^a^	1.52 ± 0.16^a^	1.61 ± 0.62^a^	1.59 ± 0.43^a^	1.82 ± 0.66^a^	ns	
Small intestines length (mm)	711 ± 57.9^a^	783 ± 55.5^a^	764 ± 74.6^a^	703 ± 35.8^a^	736 ± 91.0^a^	ns	
**Large intestines (g)**	1.29 ± 0.39^a^	1.38 ± 0.35^a^	1.44 ± 0.17^a^	1.29 ± 0.75^a^	1.38 ± 0.35^a^	ns	
(% body mass)	0.14 ± 0.03^a^	0.14 ± 0.03^a^	0.16 ± 0.06^a^	0.15 ± 0.05^a^	0.17 ± 0.17^a^	ns	
Large intestines length (mm)	85.0 ± 7.07^a^	86.9 ± 6.51^a^	83.1 ± 9.98^a^	78.6 ± 15.7^a^	80.0 ± 8.86^a^	ns	
**Caecum (g)**	2.07 ± 0.45^a^	1.88 ± 0.74^a^	1.88 ± 0.64^a^	1.64 ± 0.55^a^	1.69 ± 0.25^a^	ns	
(% body mass)	0.23 ± 0.03^a^	0.20 ± 0.09^a^	0.20 ± 0.08^a^	0.21 ± 0.06^a^	0.21 ± 0.07^a^	ns	

**Table:** 

		**Dietary treatments**				
**Parameters**	**Diet 1**	**Diet 2**	**Diet 3**	**Diet 4**	**Diet 5**	**Significance**
**Tibiae**						
Length (mm)	87.70 ± 22.3^a^	98.70 ± 7.60^a^	101.0 ± 11.5^a^	93.40 ± 10.5^a^	100.0 ± 5.41^a^	ns
Mass (mg)	3528 ± 461^a^	3624 ± 908^a^	3999 ± 1320^a^	3025 ± 1085^a^	3538 ± 891^a^	ns
Mass: length (mg.mm^–1^)	45.80 ± 22.1^a^	36.4 ± 7.45^a^	38.70 ± 10.1^a^	31.90 ± 8.49^a^	35.20 ± 8.00^a^	ns
**Femora**						
Length (mm)	67.60 ± 2.37^a^	74.2 ± 6.69^a^	74.70 ± 7.02^a^	65.90 ± 7.85^a^	70.70 ± 9.21^a^	ns
Mass (mg)	2613 ± 418^a^	2873 ± 889^a^	3225 ± 903^a^	2340 ± 989^a^	2755 ± 836^a^	ns
Mass: length (mg.mm^–1^)	38.60 ± 5.57^a^	38.6 ± 10.8^a^	42.50 ± 9.53^a^	34.60 ± 11.0^a^	38.20 ± 8.60^a^	ns

## Appendices

**Table A.1: Ingredient and nutrient composition of the grower diets for the Guinea fowl**

^∗^Vit A: 20,000,000.000 IU, Vit B_1_ (Thiamine): 003.000 g, Vit D_3_ (500 000): 3000,000.000 IU, Vit E (500 iu):40,000.000 IU, Vit K_3_ (43%): 003.000 g, Vit B_2_ (80%): 010.000 g, Vit B_6_ 98% (pyrod): 005.000 g, Vit B_12_ 1 g/kg (m):100.000 mg, Niacine 99.5%: 060.000 g, Choline (Chloride 60): 606.060 g, Biotin 2%: 200.000 mg, Manganese (MnSO4–31%):160.000 g, Copper (CuSO4–25.2%): 005.000 g, Cobalt (CoSO4–20%): 100.000 mg, Selenium (Na2SeO3- 4.5%): 400:000 mg, Calcium pantothenate: 020.000 g, Folic acid (96% pure): 001.000 g, Anty ox Vit Dry: 100.000 g, Zinc (Zn SO4–35%): 090.000 g, Iodide (KI 76.45%): 001.000 g, Ferrous (FeSO4–30%): 035.000 g, Limestone powder: 2647.133 g; DL-Methionine with purity of 99%, l-Lysine HCL with purity of 98,5%; Diet 1– 0% MNM CP substitution of SBM CP contribution, Diet 2 – 25% MNM meal CP substitution of SBM CP contribution, Diet 3 – 50% MNM meal CP substitution of SBM CP contribution, Diet 4 – 75% MNM meal CP substitution of SBM CP contribution, Diet 5 – 100% MNM meal CP substitution of SBM CP contribution.

**Table A.2: Ingredient and nutrient composition of finisher diets for the Guinea fowl**

∗Vit A: 20,000,000.000 IU, Vit B_1_ (Thiamine): 003.000 g, Vit D_3_ (500 000): 3000,000.000 IU, Vit E (500 iu):40,000.000 IU, Vit K_3_ (43%): 003.000 g, Vit B_2_ (80%): 010.000 g, Vit B_6_ 98% (pyrod): 005.000 g, Vit B_12_ 1 g/kg (m):100.000 mg, Niacine 99.5%: 060.000 g, Choline (Chloride 60): 606.060 g, Biotin 2%: 200.000 mg, Manganese(MnSO4–31%):160.000 g, Copper (CuSO4–25.2%): 005.000 g, Cobalt (CoSO4–20%): 100.000 mg, Selenium (Na2SeO3- 4.5%): 400:000 mg, Calcium pantothenate: 020.000 g, Folic acid (96% pure): 001.000 g, Anty ox Vit Dry: 100.000 g, Zinc (Zn SO4 – 35%): 090.000 g, Iodide (KI 76.45%): 001.000 g, Ferrous (FeSO4 – 30%): 035.000 g, Limestone powder: 2647.133 g; DL-Methionine with purity of 99%, l-Lysine HCL with purity of 98,5%; Diet 1 – 0% MNM CP substitution of SBM CP contribution, Diet 2 – 25% MNM meal CP substitution of SBM CP contribution, Diet 3 – 50% MNM meal CP substitution of SBM CP contribution, Diet 4 – 75% MNM meal CP substitution of SBM CP contribution, Diet 5 – 100% MNM meal CP substitution of SBM CP contribution.

**Table A.3: Effect of graded dietary substitution of Soyabean meal with Marula nut meal on the growth performance and feed utilisation efficiency of broiler Guinea fowl** ns = not significant, **P* < 0.05, ^⁎⁎^*P* < 0.01, ^a^ Within row means with the same superscripts are not significantly different (*P* > 0.05). Guinea fowl fed diet 3 (50% MNM) had a significantly high BMG and ADG in week 2 of the experiment compared to other treatments. MNM had no significant effect (*P* > 0.05) on the birds’ overall body weight gain, ADG (weekly and overall), FI throughout all the dietary treatments. Overall FCR was significantly (*P* < 0.05) higher for birds fed 50% (Diet 3) MNM inclusion than other dietary treatments. BMG- body mass gain, ADG - average daily gain, FI - feed intake, FCR - feed conversion ratio Diet 1– 0% MNM CP substitution of SBM CP contribution, Diet 2 – 25% MNM meal CP substitution of SBM CP contribution, Diet 3 – 50% MNM meal CP substitution of SBM CP contribution, Diet 4 – 75% MNM meal CP substitution of SBM CP contribution, Diet 5 – 100% MNM meal CP substitution of SBM CP contribution. Data presented as mean ± SD; *n* = 7 to 8.

**Table A.4: Effect of dietary substitution of Soyabean meal with Marula nut meal on gastrointestinal organ and accessory organ masses and lengths of Guinea fowl** n.*s* = not significant, P ˃ 0.05. ^a^ Within row means with the same superscripts are not significantly different (*P* > 0.05). The viscera weights and relative body mass were statistically similar (P ˃ 0.05) for Guinea fowl across dietary treatments. Diet 1– 0% MNM CP substitution of SBM CP contribution, Diet 2 – 25% MNM meal CP substitution of SBM CP contribution, Diet 3 – 50% MNM meal CP substitution of SBM CP contribution, Diet 4 – 75% MNM meal CP substitution of SBM CP contribution, Diet 5 – 100% MNM meal CP substitution of SBM CP contribution. Data presented as mean ± SD; *n* = 7 to 8.

**Table A.5: Effect of graded dietary substitution of Soyabean meal with Marula nut meal on tibiae and femora length, mass, and bone mass to length ratio of broiler Guinea fowl** ns = not significant, P ˃ 0.05. Femora lengths, masses and masses to length ratio of the Guinea fowl across dietary treatments were similar (P ˃ 0.05). Diet 1– 0% MNM CP substitution of SBM CP contribution, Diet 2 – 25% MNM meal CP substitution of SBM CP contribution, Diet 3 – 50% MNM meal CP substitution of SBM CP contribution, Diet 4 – 75% MNM meal CP substitution of SBM CP contribution, Diet 5 – 100% MNM meal CP substitution of SBM CP contribution. Data presented as mean ± SD; *n* = 7 to 8.
